# Machine learning methods for detecting urinary tract infection and analysing daily living activities in people with dementia

**DOI:** 10.1371/journal.pone.0209909

**Published:** 2019-01-15

**Authors:** Shirin Enshaeifar, Ahmed Zoha, Severin Skillman, Andreas Markides, Sahr Thomas Acton, Tarek Elsaleh, Mark Kenny, Helen Rostill, Ramin Nilforooshan, Payam Barnaghi

**Affiliations:** 1 Department of Electrical and Electronic Engineering, Centre for Vision, Speech and Signal Processing (CVSSP), University of Surrey, Surrey, United Kingdom; 2 Surrey and Borders Partnership NHS Foundation Trust, Leatherhead, Surrey, United Kingdom; University of Murcia, SPAIN

## Abstract

Dementia is a neurological and cognitive condition that affects millions of people around the world. At any given time in the United Kingdom, 1 in 4 hospital beds are occupied by a person with dementia, while about 22% of these hospital admissions are due to preventable causes. In this paper we discuss using Internet of Things (IoT) technologies and in-home sensory devices in combination with machine learning techniques to monitor health and well-being of people with dementia. This will allow us to provide more effective and preventative care and reduce preventable hospital admissions. One of the unique aspects of this work is combining environmental data with physiological data collected via low cost in-home sensory devices to extract actionable information regarding the health and well-being of people with dementia in their own home environment. We have worked with clinicians to design our machine learning algorithms where we focused on developing solutions for real-world settings. In our solutions, we avoid generating too many alerts/alarms to prevent increasing the monitoring and support workload. We have designed an algorithm to detect Urinary Tract Infections (UTI) which is one of the top five reasons of hospital admissions for people with dementia (around 9% of hospital admissions for people with dementia in the UK). To develop the UTI detection algorithm, we have used a Non-negative Matrix Factorisation (NMF) technique to extract latent factors from raw observation and use them for clustering and identifying the possible UTI cases. In addition, we have designed an algorithm for detecting changes in activity patterns to identify early symptoms of cognitive decline or health decline in order to provide personalised and preventative care services. For this purpose, we have used an Isolation Forest (iForest) technique to create a holistic view of the daily activity patterns. This paper describes the algorithms and discusses the evaluation of the work using a large set of real-world data collected from a trial with people with dementia and their caregivers.

## Introduction

There are currently around 46.8 million people living with dementia around the world, and this number is estimated to increase to 74.7 million by 2030 and to 131.5 million by 2050 [1]. Generally, people with dementia have a greater risk of hospitalisation compared to people of the same age without dementia, approximately about 40% of elderly patients admitted to general hospitals in the UK have dementia [2–4]. In the UK, people with dementia occupy around 20% of general hospital beds and have increased length of stay with higher re-admission rate (two to three times more often than people of the same age without dementia) [2, 4, 5]. Although hospital admissions are more frequent in people with dementia, the number of patients admitted to hospital for dementia as the ‘primary diagnosis’ is very low (on average, it is about 7% for all types of dementia) [6]. Therefore, several studies have focused on specific factors leading to hospital admission for people with dementia [3, 6–10]. According to the literature, the primary risk factors are divided into two main categories: (i) physical health-related factors and (ii) psychiatric factors, where both categories are associated with increasing severity of cognitive impairment and dementia [3].

- Physical health-related factors: According to the literature, people with dementia are at increased risk of some specific physical health-related factors, including orthopaedic, respiratory, urology/renal, gastrointestinal, and cardiology conditions [3, 6, 7]. Among which, the most prevalent causes of hospitalisation include urinary tract infection (UTI), chest infection, falls, and hip fracture/replacement [2, 6, 7, 11–13].
- Psychiatric factors: In addition to the physical health-related factors, the psychiatric and behavioural factors were also found to increase the risk of hospital admission for people with dementia compared to those without dementia and in the same age group [7]. The behavioural and psychological symptoms of dementia (BPSD) consists of seven domains: paranoid and delusional ideation, hallucinations, activity disturbances, aggressiveness, daily rhythm disturbance, affective disturbance, and anxieties and phobias—among which aggression, activity disturbance and sleep disturbance are the most common symptoms [4]. In particular, it was shown that behavioural disturbances, such as agitation, wandering and sleep disorder [7, 9–11], and changes in routine and environment [7, 14] are associated with a higher risk of hospitalisation for people with dementia—where sleep disorder was identified as the most significant factor [11]. Therefore, analysing the daily routine and observing the night-time sleep pattern play an important role in monitoring the health and well-being of people with dementia.

According to previous studies [2, 7] and a recent report from the UK National Audit Office [15], people with dementia may receive poorer quality of care and less positive outcomes in acute hospitals and this can have adverse effects on symptoms of dementia. Therefore, it is essential to reduce (and ideally avoid) unnecessary hospital admissions. This can be achieved through early detection of problems and appropriate interventions by multidisciplinary teams, including medical staff, social workers and/or trained care staff [7, 16]. In this regard, we have developed an advanced Internet of Things (IoT) platform to propose a Technology Integrated Health Management (TIHM) system for people with dementia. TIHM is a technology assisted monitoring system that uses different, yet complementary, IoT-enabled technologies for continuous monitoring of people with dementia in their own homes. The collected data in the TIHM project is integrated and processed using several data analytics and machine learning algorithms to generate personalised notifications based on patients’ needs, aligned to the parameters set by clinicians. This information is monitored around the clock by a group of healthcare practitioners who take appropriate decisions by following a clinical pathway taking into account the collected data and actionable information. This facilitates an automated monitoring system which assists the healthcare practitioners to use the information provided to inform clinical judgement, provide more effective support and ensure that the right level of support can be deployed at the earliest point of need to prevent escalating crises.

In order to provide actionable information, with the support of clinical experts, we have co-designed machine learning and data analytics algorithms that combine environmental and physiological data to learn and discover changes in patients’ health and well-being. These algorithms are divided into two main categories (i) adaptive algorithms for personalised thresholding and (ii) learning algorithms for pattern analysis. The first set of algorithms are mainly designed to analyse vital measurements and develop personalised adaptive thresholds based on the distribution of individuals’ historical data; whereas, the latter set of algorithms are designed to analyse the combination of physiological and environmental data to learn the participants’ activities/patterns [16, 17]. For instance, our previous study [18] focused on an algorithm for analysis of higher-level of activity patterns to detect any change in patients’ routines and a hierarchical information fusion approach for detecting agitation, irritability and aggression. In addition, given the importance of specific physical and psychiatric factors in hospitalization of people with dementia, this study focuses on the detection of UTI and analysis of daily routine, including the sleep patterns—which are discussed in sections ‘Urinary tract infection’ and ‘Health and well-being factors’.

This paper is structured as follows. We first focus on the study design to clarify the trial procedure, followed by the purpose statement to highlight the two risk factors studied in this work (including UTI and daily pattern analysis). We then provide the related work section to discuss the existing works in this area, followed by our proposed methodologies to analyse/detect the above risk factors. We evaluate the proposed algorithms and discuss the results. The last section provides a conclusion and briefly explains the directions for future work.

## Study design

Participants in this study have had a confirmed diagnosis of dementia (mild to moderate) within, at least, the past three months and have been stable on dementia medication. Participants live in the Surrey county in the UK and have been receiving a minimum of 10 hours care per week from their family or a formal caregiver. Each participant was recruited for six months trial period. During the trial, the environmental data was continuously recorded via sensors installed in participants’ homes. The environmental sensors consist of 2 passive infra-red (PIR) sensors (installed in the hallway and living room), 4 motion sensors (one in the kitchen, one on the pill box/drawer and two on the bedroom and bathroom doors), 2 pressure sensors (deployed on the bed and the chair), 1 main entrance door sensor and 1 central energy consumption monitoring device. The physiological data was recorded twice a day by participants using the Bluetooth-enabled medical devices. The physiological data includes blood pressure, heart rate, body temperature, weight and hydration readings. All the sensory devices are provided by technology partners in the project listed at https://www.sabp.nhs.uk/tihm/about/our-partners.

The study protocol for this work has been reviewed and approved by the South East Coast Surrey NHS Research Ethics Committee. We obtained informed consent from all the study participants. Each participant was assessed according to Mental Capacity Act guidelines by a fully qualified researcher who has completed a mandatory clinical practice course. Participants understood the study and were able to understand the consent process.

## Purpose statement

In this paper, we focus on two risk factors which capture both physical health-related and psychiatric symptoms. To this end, we use the collected data to develop algorithms for detecting UTI and analysing daily living activities, which are discussed below.

### Urinary tract infection

Given that UTI is one of the most commonly diagnosed infections in older adults accounting for a high number of visits to healthcare providers [12, 13, 19], we have developed an algorithm to detect the possibility of UTI using in-home sensory data. Early identification of UTI is particularly crucial for older adults as delayed treatment invites further complications and can be catastrophic. An early assessment of UTI symptoms does not only provide an opportunity to intervene early in order to enable appropriate treatment, but it can also be used to identify the likely cause of other developing problems such as confusion, agitation or behavioural shifts. Since these symptoms are typically common for people with dementia, the possible underlying cause is often overlooked and this results in the late diagnosis and treatment of UTI.

Recently, pervasive technologies hold the promise of developing objective, real-time, and continuous measurement methodologies that were previously infeasible. Such technologies can greatly contribute to a deeper understanding of the activity and behavioural patterns of individuals. In this regard, the application of machine learning algorithms to identify behavioural anomalies is the first step toward personalised symptom management. Similarly, one of the goals of this study is to develop an algorithm for the early detection of abnormal health conditions associated with UTI in order to initiate symptom management intervention prior to the initiation of treatment.

### Daily pattern analysis

Inspired by existing literatures on the importance of specific psychiatric factors for hospitalisation of people with dementia [7, 9–11, 14], we have focused on analysis of daily routines and night-time sleep patterns of people with dementia using in-home sensory data.

To date, several studies have focused on the activity recognition and routine detection using in-home sensors where some demonstrated the detection of cognitive changes using environmental sensors, such as motion, pressure, and door sensors [20–23]. In particular, [4] and [23] showed that analysis of daily routine plays an important role in monitoring the health and well-being of people with dementia which can help identify their behavioural changes. In the same spirit, we have developed machine learning algorithms that provide longer-term information about the participants’ routines (by analysing their daily activities inferred from sensory observations) and identify if they are constantly deviating from their routine, i.e. exhibiting unusual patterns (‘anomalies’).

## Related work

This section provides an overview of the related work that use environmental and/or physiological markers to extract information for managing conditions or avoiding adverse health conditions. We first review the UTI related work and then discuss the analysis of daily living activities.

### Urinary tract infection

The gold standard for diagnosis of UTI is detection of the pathogen in the presence of clinical symptoms. The pathogen is detected and identified by urine culture (using midstream urine). However, due to high diagnostic error, earlier research efforts are mainly focused on improving the diagnostic accuracy of the predictive models that incorporate multiple aspects of historical, physical, and laboratory findings [24–26]. Note that such approaches are reactive as they require clinical interventions and a priori knowledge of the infection risks which often result in delayed treatment; whereas, early detection of UTI symptoms can be used as a proactive solution for early intervention when the treatment is most effective. This can be made possible by enabling the deployment of monitoring systems that continuously monitor the day-to-day activities of the elderly in a (near) real-time manner to capture the physiological parameters. Motivated by this, our study is focused on early detection of UTI symptoms in people with dementia using a set of in-home sensors and employing machine learning algorithms.

In recent years, there is a growing interest in the use of Ambient Assisted Living (AAL) technologies aimed at improving the monitoring of elderly people to enhance their quality of life [20–22, 27]. In order to monitor the health status of elderly people, their activity patterns have been studied by installing a variety of sensors (e.g. motion sensors, discrete switches and video cameras) within individuals’ homes. The information has been combined with physiological measurements, including but not limited to blood pressure, body weight and heart rate, for the detection of cognitive changes. For example, “Welfare Techno House” project [28] profiles health status of the occupant by measuring electrocardiograph (ECG), body weight and urinary volume using sensors placed in the bathroom. Likewise, researchers at Centre for Advanced Studies in Adaptive Systems (CASAS) focus on detecting a drift in the everyday routine of the occupant by capturing his/her activities and movement patterns with the use of PIR, kinetic, and door sensors [29]. Similarly, there have been several studies that capture and investigate motion density and movement patterns of elderly population to discover and understand physical, cognitive and perceptual decline [30–32]. However, much of the research is specifically focused on cognitive decline and there is less work on detecting early health changes for general health management.

Early detection of health symptoms is further complicated due to the variety of home configurations and the fact that many seniors have multiple chronic health conditions. In this study, we make use of in-home PIR sensor data to extract Time Sensitive movement Patterns (TSP). These patterns are used to detect changes in occupants’ routine which can be generalised to different chronic health conditions. The TSP in conjunction with the body temperature of the occupant is used to develop an early UTI detection model. A similar work [30] has been done which aimed to detect UTI symptoms using in-home PIR sensors. However, the proposed method was based on supervised learning approach to develop and discover patterns, which depended on the activity labels and annotations in the training data set. For almost all of the smart home testbeds, the data collection and data labelling are two separated processes. The activity labelling process is extremely time consuming since it is usually based on manual annotation efforts. Such limitation makes it difficult to generalise corresponding algorithms to the real-world situations with huge amount of unlabelled data collected from uncontrolled environments. Thus, in this paper, we build a system in order to discover and learn the daily activity patterns of the participants in an unsupervised fashion. More specifically, we present an unsupervised technique to learn individual’s movement patterns directly from the unlabelled PIR sensor data.

**Fig 1:**
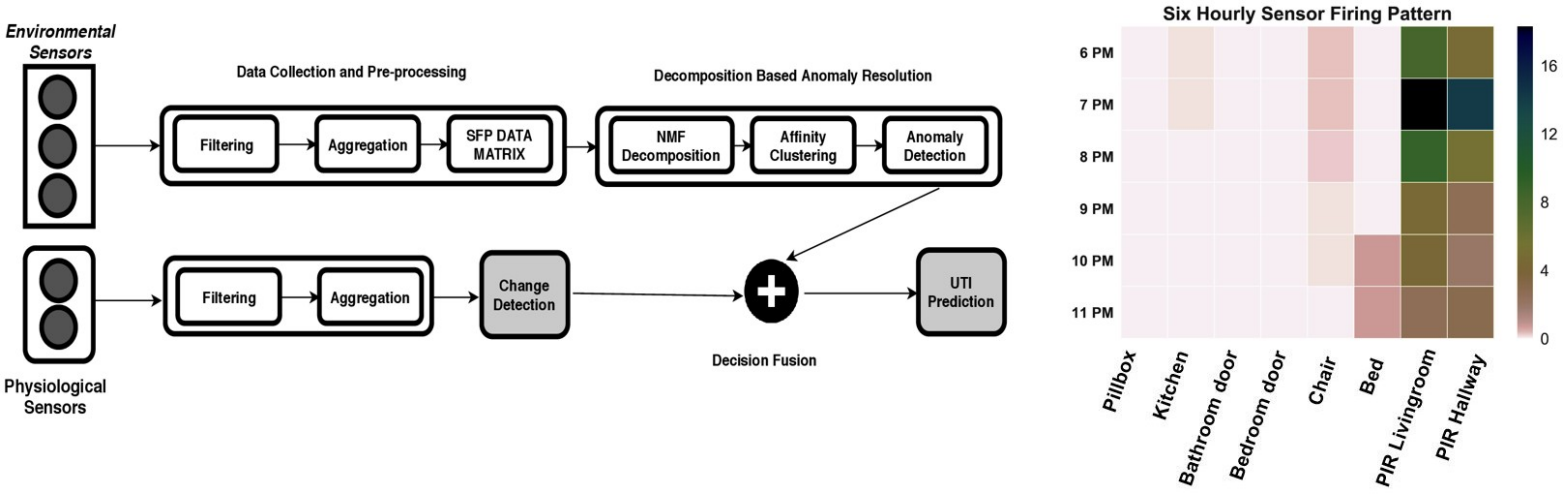
An unsupervised framework for early UTI prediction (left) a visualisation of six-hour Sensor Firing Pattern (SFP) data matrix (right).

A high-level structure of the proposed method has been shown in Fig 1 (left) and is further discussed in Section ‘Unsupervised early detection model’. Furthermore, in order to provide a comparison to our proposed approach, a one-class support vector machine (SVM) model has been trained using a clinically approved annotated dataset, the details of which has been provided in Section ‘One-class support vector machine model’.

### Daily pattern analysis

In our previous work [18], anomalies were defined based on the sequence of activities where the sequence alignment was used to identify unusual pattern in daily activities. We used *Markov chains* to model the activity sequences and then calculated an *entropy rate* to quantify the regularity of individuals’ patterns in their day-to-day life. According to [23] and [33], people with dementia exhibit one or more symptoms which may occur at any time of the day, making them have more non-structured routine compared to healthy individuals. This means that the proposed entropy-based algorithm, which focused on the analysis of daily sequences, might not be an optimal approach for pattern analysis of people with dementia. Therefore, in this paper, we define anomalies based on a holistic view of daily activities, rather than their sequences. Each day is characterised by a map of aggregated daily activities and those that have different pattern characteristics from normal instances are defined as anomalies, see Section ‘Isolation Forest Algorithm’ for full details.

The anomalies detected through the above approach associate with different scenarios, for instance, technical issues (e.g. connectivity problems or sensor malfunctioning), temporary changes in the recording environment (e.g. being away for multiple days or having visitors), health conditions (e.g. more frequent bathroom visit due to infection) and/or personal activity patterns (e.g. being agitated or restless more often). In our previous study [18], we did not provide any complementary information about the detected anomaly, i.e. we focused on the detection of anomalies regardless of the reason of their origins. Furthermore, we did not analyse the night-time sleep patterns of participants; while, according to the literature [4, 10, 11], sleep disorder has been identified as one of the most significant psychiatric factors for hospitalisation of people with dementia. Therefore, in this study, we monitor the sleep pattern, health status, and technical/environmental conditions in parallel to the anomaly detection algorithm to extract meaningful insight about the possible cause(s) of anomalies, see Section ‘Health and Well-being Factors’.

## Methodology

In our work, we have implemented an unsupervised machine learning algorithm for UTI detection and compared it with a supervised model for the same purpose. We have also used an Isolation Forest algorithm for analysing daily living activities. These methods are discussed below.

### Unsupervised early detection model

The UTI detection algorithm uses physiological and environmental observations obtained from participants’ homes to detect anomalous patterns that are correlated with UTI symptoms. Based on sensory data, our proposed algorithm discovers the normal and abnormal activity patterns of individuals in an unsupervised manner. Subsequently, it correlates the extracted information with physiological data to predict an abnormality in the health condition.

#### Data collection and pre-processing

In our study, we have collected environmental and physiological measurements to extract actionable information about an individual’s activities, trends and routines. More specifically, to analyse the activity patterns of participants, we have selected eight view points (instead of ten) for the UTI algorithm, i.e. ignoring the door sensor and central energy consumption monitoring device from the list stated in Study design. Since during our initial investigation we found out that readings from the door sensor and central energy consumption monitor are highly sensitive to multi-occupancy behaviour within a home and are not helpful for decoupling participants’ activity pattern. For example, due to presence of guests, door sensor results in repetitive readings that mostly act as an outlier. Likewise, the central energy consumption monitoring device monitors the overall energy consumption of the home and therefore provides an aggregated energy usage pattern of the occupants.

The data pre-processing module collects information from each viewpoint, filters and subsequently aggregates the readings within a time interval of one hour. This is followed by concatenating the hourly readings of each viewpoint to generate an hourly Sensor Firing Pattern (SFP). The hourly SFPs are then grouped together in a time interval of 6 consecutive hours to generate a six-hour view of participants’ activities. A visualisation of six-hour SFP data matrix has been shown in Fig 1 (right). The six-hour SFP data matrix serves as an input sample to our UTI detection algorithm. The repeatability of the activity patterns are highly correlated with the time of the day. The main advantage of analysing participants’ patterns in a six-hour activity window is that SFPs can be easily analysed within four distinct time categories: morning (06:00AM to 12:00PM), afternoon (12:00PM to 6:00PM), evening (6:00PM to 0:00AM) and night (0:00AM to 6:00AM); where these six-hour views are stored in the database for further processing.

#### Non-negative matrix factorisation for pattern extraction

In the profiling phase, the UTI detection algorithm analyses three months of participant’s data by generating corresponding six-hour SFP views for each time category. In order to learn unique views that occur repeatedly within the participant’s data for each time category, we have applied Non-negative Matrix Factorisation (NMF) algorithm to extract latent factors. NMF is a technique for linear dimensionality reduction that yields a part-based, sparse, non-negative representation for non-negative input data. In our case, the input data is the six-hour SFP data matrix *V*. NMF is an unsupervised learning algorithm, originating from linear algebra, that not only reduces data dimensionality, but also performs clustering simultaneously. NMF seeks to decompose a data matrix *V* into the product *W* × *H* where *W*_*m*×*r*_ is the matrix of features and *H*_*r*×*n*_ is the matrix of coefficients. Usually *r* is chosen to be smaller than either *m* or *n*, for dimensionality reduction. Thus, each column of *V* is approximated by a linear combination of the columns of *W* with coefficients being the corresponding column of *H*, as shown in Eq (1):

$$\begin{array}{c} v_{j}\approx \sum_{i=1}^{r}w_{i}h_{i,j} \end{array}$$ (1)

where the decomposition is created by solving the following non-linear optimisation problem:

$$\begin{array}{c} {\min∥V-WH∥}_{F}^{2}\;\;\text{such}\;\text{that}\;W,H\geq 0 \end{array}$$ (2)

The NMF solutions are not unique since most NMF algorithms perform random initialisation of *W* and *H* matrices. We have used Alternating Constrained Least Squares (ACLS) method [34] in our experiments. Interested readers can refer to [34] for more information about ACLS. This method extracts underlying features of the data as basis vectors (*w*_*i*_) which can then be used for identification, clustering, and data compression.

The decomposition of SFP data matrix reveals the latent factors underlying the interactions between hour of the day and the activation sequence of the sensors. Rather than clustering the data into groups according to the dominant feature vector associated with each point, we proposed to cluster the data according to their coordinates in the lower dimensional “feature space”. In doing so, we allow more features in data than there are clusters (unlike the conventional NMF clustering algorithm which requires the number of features found by the decomposition to be equal to the number of desired clusters). The proposed algorithm for profiling SFPs of participants has been shown in S1 Algorithm.

The clusters obtained by S1 Algorithm represent distinctive SFPs obtained from the user data. Note that the number of total clusters *k* has been determined by applying the silhouette coefficient criteria [35] and the number of SFPs within each cluster is an indication of their repeatability.

#### Anomaly detection

Once the total number of clusters has been obtained, the next step is to perform a top-level categorisation of these clusters into three distinct categories: highly repetitive SFPs (HSPF), low repetitive SFPs (LSPF), and rarely repetitive SFPs (RSPF). To achieve this, an array is populated based on the count of SFPs within each cluster. As shown in S2 Algorithm, this array is then used to compute a Median Absolute Deviation (MAD) score, the details of which are provided in [36]. A MAD score is used to measure the dispersion of a univariate quantitative data. It provides the deviation or variability of the data point around the median value. A deviation score is calculated for each data point as shown in S2 Algorithm. This score can then be used to identify or filter out anomalies from the data using different rejection criterion.

We have investigated three different rejection criterion for the S1 Algorithm: 3 (very conservative), 2.5 (moderately conservative) and 2 (poorly conservative) as proposed in [32]. The threshold selection of 2.5 has been made empirically by analysing the patterns that has been categorised as RSRP (i.e., a category of outlying patterns) by different rejection values. Likewise, samples within one standard deviation of the MAD are considered to be normal and therefore a threshold value of 1 is chosen to categorise patterns into HSFP (i.e., category containing normal patterns). Therefore, in our experimental analysis, the rejection criteria *D* has been found out to be 1 and 2.5 and is used to assign each data point to one of the three SFP categories; i.e. the clusters with scores less than 1 belongs to HSFP, whereas the ones that have obtained a score of greater than 1 but less than 2.5 are assigned to LSFP. Finally, any cluster that has a score of 2.5 or above is labelled as RSFP category.

The HSFP category contains highly repetitive SFPs, whereas LSFP contains patterns for which the repeatability probability is less than the patterns in HSFP. The patterns obtained within the HSFP category profiles the normal behaviour of the participant. Likewise, the patterns within LSFP can reveal the patterns with less occurring frequency, e.g. staying-up late during the weekends. Finally, the RSFP category contains those SFPs that represent an anomaly in the participant’s pattern, since these patterns have very low probability of occurrence. For example, it is highly unlikely for a participant to be moving around the house throughout the night or there has been a significant change in the bathroom visit frequency during the night. Therefore, the RSFPs can be correlated with clinical measurements to infer any possible health issue as discussed below. The anomaly detection block generates an alert and feeds it to the decision fusion block, in case a real-time SFP data matrix is categorised into an RSFP.

#### Decision fusion

As for clinical measurements, each participant is required to take a body temperature measurement which is automatically forwarded to the back-end system. According to [37], a change in the body temperature is considered as strong predictive indicators for infection. The increase in the frequency of bathroom activity in conjunction with body temperature greater than 38 Celsius are among symptoms of a possible UTI. As shown in Fig 1 (left), the change detection module generates an alert only if the body temperature has increased beyond a specified threshold and feeds it to the decision fusion block. The decision block outputs a UTI alert only if it has received an increase in the body temperature alert and the RSFP alert within a time window of 24 hours.

### One-class support vector machine model

In order to compare the proposed unsupervised UTI detection model with a supervised method, we have also trained a one-class SVM model [38]. The one-class SVM model serve as a baseline model and therefore we have decided to rely only on those indicators which are clinically accepted and are well studied in literature. Our baseline model takes an input of participants’ body temperature and frequency of bathroom visit information to predict UTI infection. As highlighted earlier, the change in the body temperature and increase in the urination are two strong indicators [37] exhibited by patients diagnosed with UTI. Moreover, our one-class SVM model has been trained on manually annotated dataset (annotated by expert clinicians) that contains the following information about patients diagnosed with UTI: frequency of the bathroom visit and body temperature. These two variables are subsequently used to train and determine the decision boundary of the one-class model. The one-class SVM model maps the input data to high-dimensional feature space using Kernel trick [38] and iteratively finds a maximum margin hyper-plane (i.e., or a decision boundary) via an optimisation algorithm that separates the training data from origin. The learned decision boundary is then used to classify unseen data points. Once the model has been trained, it has been tested on the real-time data obtained from the TIHM project. The results and performance analysis are reported in Section ‘Results and Discussions’. Besides the UTI detection algorithm, we have developed an algorithm to analyse individual’s daily pattern and detect anomalies which is discussed below.

### Isolation forest algorithm

Unlike our previous study, where anomalies depended on the *sequence* of daily activities, in this paper, anomalies are defined based on the *holistic view* of daily activities rather than their sequences. To this end, days are characterised by aggregated daily activities and those with very different characteristics, according to the anomaly detection algorithm, are defined as anomalies.

The environmental data used for the iForest algorithm is collected from a set of passive sensors installed in participants’ homes. We have used 10 sensors in total, as described in the Study design [18]. The data is collected from multiple sources deployed in uncontrolled real-world environments, i.e. private homes. This means that data may be noisy and incomplete (containing missing values) with various sampling rates for different sources. Therefore, the raw data is first pre-processed and aggregated over one-hour intervals; i.e. each day is represented by 24 windows and each window is an array of *N* elements where *N* is the number of nodes for each participant. Furthermore, to perform feature scaling, the aggregated data is normalised to ensure that the activity levels of all sensors are represented within the same range [0-1] and equally taken into consideration for further analysis. A set of 30 days is used as the training data and fed to the iForest algorithm to construct a personalised estimator model for each participant. The same model is then used to label a test data as either normal or abnormal.

### Health and well-being factors

The anomalies detected via iForest algorithm may associate with different scenarios and further investigation is required to identify whether these deviations are related to the technical infrastructure faults, environmental changes or associated with possible health and well-being conditions which may indicate the progression of dementia in participants. Therefore, in this work, we monitor these factors (i.e. technical, environmental, health and well-being) to extract actionable information in parallel to the anomaly detection algorithm. In this paper, we only describe some of the health and well-being factors, such as night-time sleep pattern and the daily health score (DHS)—which are discussed below.

#### Sleep pattern analysis

Irregular sleep pattern is one of the general symptoms experienced by people with cognitive impairment, including those who have dementia [39]. In particular, sleep disorder is one of the most significant psychiatric factors of hospital admission for people with dementia, compared to those without [11]. Therefore, it is advantageous to monitor and assess the sleep pattern of elderly with dementia. To date, several approaches have been used for monitoring the sleep pattern, including personal diary, polysomnography, wearable technologies and passive assistive technologies.

The personal diary includes reports from patients and/or their caregivers; although this is an easy and low-cost approach, it is subjective and might be inaccurate due to human error [40, 41]. The second approach, i.e. polysomnography (PSG), uses a combination of electroencephalogram (EEG), electrooculogram (EOG), and electromyogram (EMG) to detect electrical activity in the brain, monitor eye movements, and measure muscle activities, respectively [42]. Although this approach provides detailed and accurate information about sleep quality, it requires medical devices and professional skills and is not suitable for in-home monitoring. The next approach, also known as actigraphy [43], monitors activity and identifies sleep stages using an accelerometer embedded in a wearable device (e.g. watch) [44, 45]. Though easier than the PSG approach, these products still require a device to be attached to the individual’s wrist and their measurements are often not very accurate. On the other hand, the next approach exploits a set of passive technologies (e.g. motion and occupancy sensors) to remotely monitor sleep pattern in homes, providing an easy, objective, cost-effective and ubiquitous approach [46]—which is also used in the TIHM for dementia project. To date, several studies have focused on analysis of sleep pattern for people with dementia [39, 46–48]. However, these studies involved a small number of participants and they only considered those who were living independently [39]. Whereas, our TIHM project (so far) involved 82 participants living in their own homes, without any occupancy restriction, as the majority cohabit with spouses or other family members. In addition, the combination of bed pressure sensor and further environmental sensors captures both bed activity, as well as the movement activities at night that occur around the house.

In this project, we analyse the night-time sleep pattern from two aspects: sleep duration and sleep quality, i.e. duration of restlessness and wandering. We integrate the data collected from ten passive sensors and apply a two-level rule-based reasoning algorithm [49] to identify the sleep patterns. The first set of rules only consider the bed pressure sensor to pre-process the raw data and extract definitive in-bed and out-of-bed epochs. The second set of rules are then applied on the integrated data to identify semantic instances/states of interest, such as ‘bed entry’, ‘sleep onset’ and ‘sleep offset’ instances and ‘sleep (good or moderate)’, ‘restless sleep’, ‘wandering’, and ‘out of bed’ states. To analyse the night-time sleep continuously, the night hours is defined from 18:00 to the next day 12:00 [39, 48] to (reasonably) be assured that participant is out of bed. During this period, transition instances of the bed sensor are recorded as *o*_*i*_ and *ϕ*_*i*_, representing instances that the bed sensor is detected as occupied (activated) and empty (deactivated), respectively. The raw data (**X**) is pre-processed using the first set of rules defined in S3 Algorithm (top) to filter the short-time triggers (e.g. turning over in bed) and extract definitive in-bed and out-of-bed epochs, given by:

$$\text{X}=\begin{array}{c} \begin{array}{llll} \text{On} & \text{Off} & \text{Length} & \text{Gap} \end{array}\\ \left[\begin{array}{cccc} o_{1} & \phi_{1} & \tau_{1} & \delta_{1}\\ o_{2} & \phi_{2} & \tau_{1} & \delta_{2}\\ \vdots & \vdots & \vdots & \vdots \\ o_{i} & \phi_{i} & \tau_{i} & \delta_{i} \end{array}\right] \end{array}\;\to \;\hat{\text{X}}=\begin{array}{c} \begin{array}{llll} \text{On} & \text{Off} & \text{Length} & \text{State} \end{array}\\ \left[\begin{array}{cccc} {\hat{o}}_{1} & {\hat{\phi }}_{1} & {\hat{\tau }}_{11} & \text{in}\\ {\hat{\phi }}_{1} & {\hat{o}}_{2} & {\hat{\tau }}_{12} & \text{out}\\ {\hat{o}}_{2} & {\hat{\phi }}_{2} & {\hat{\tau }}_{22} & \text{in}\\ {\hat{\phi }}_{2} & {\hat{o}}_{3} & {\hat{\tau }}_{23} & \text{out}\\ \vdots & \vdots & \vdots & \vdots \\ {\hat{o}}_{i} & {\hat{\phi }}_{i} & {\hat{\tau }}_{ii} & \text{in} \end{array}\right] \end{array}$$ (3)

in which *τ*_*i*_ = *ϕ*_*i*_ − *o*_*i*_ is the duration of an occupied epoch and *δi* = *o*_*i*+1_ − *ϕ*_*i*_ is the gap between two consecutive occupied epochs in the raw data **X**. On the other hand, ${\hat{\tau }}_{ii}$ and ${\hat{\tau }}_{ij}$ (for *i* ≠ *j*) represent duration of definitive in-bed and out-of-bed epochs after the initial pre-processing. The pre-processed data $\hat{X}$ is then fed to the the second set of rules in order to annotate the states of interest. We have developed a temporal rule-based reasoning algorithm using the sleep stages defined in the American Academy of Sleep Medicine (AASM) [50]. According to the AASM, sleep is divided into two broad classes known as rapid eye movement (REM) and non-rapid eye movement (NREM) which itself is further divided into three distinct stages N1, N2, and N3. A sleep cycle in adults lasts between 90 to 100 minutes and it begins with three stages of NREM followed by REM, i.e. N1-N2-N3-REM. The first stage (N1) is a transition from wakefulness and it refers to the lightest and shortest stage of sleep with duration 1-7 minutes. In the next stage (N2), which lasts between 10 to 25 minutes, the body reaches a state of relaxation in preparation for the deep sleep. After N2, an adult enters the deep sleep stage (N3) which lasts from 20 to 40 minutes. Following the N3, an adult ascends to lighter NREM sleep again, before entering the REM sleep episodes [50, 51]. Given this information, we define a temporal rule-based reasoning algorithm to analyse the pre-processed sleep data ($\hat{X}$) along with the integrated data from other environmental sensors (including PIR, motion, door, and chair pressure sensors) to label sleep blocks as ‘sleep (good or moderate)’, ‘restless sleep’, ‘wandering’, and ‘out of bed’ states, see S3 Algorithm (below).

Note that temporal thresholds used in S3 Algorithm are set based on the given minimum and maximum expected duration of sleep stages *N*_*i*_, representing as *Ni*_L_ and *Ni*_H_, respectively (*i* ∈ {1, 2, 3}). For instance, since N1 lasts between 1 to 7 minutes, the minimum and maximum expected duration of N1 is defined as *N*1_L_ = 1 and *N*1_H_ = 7. Accordingly, the sleep on-set instance and restless sleep state are defined based on the duration of N1 and N2 stages. For example, sleep on-set is the first in-bed epoch which lasts more than the average duration of *N*1 + *N*2, given by *τ*_*ii*_ > mean(*N*1_L_ + *N*2_L_, *N*1_H_ + *N*2_H_) = mean(1 + 10, 7 + 25), and restless sleep is defined as a sleep epoch which lasts less than the minimum duration of *N*1 + *N*2, given by *τ*_*ii*_ < *N*1_L_ + *N*2_L_ = 1 + 10, and so on.

Quantifying the length and quality of nocturnal sleep depends on a great number of factors, differing from person to person and from night to night, and it is thus difficult to characterise a generic ‘normal’ pattern [51]. To resolve this, we analyse the historical sleep pattern of individuals for 30 days to define adaptive personalised deviation boundaries for minimum duration of sleep and maximum duration of restlessness/wandering. Accordingly, any test data exceeding these thresholds is detected as abnormal sleep pattern, i.e. sleep disturbance.

#### Daily health score

As discussed, it is important to understand the causality of anomalies detected via iForest algorithm; especially those associated with health and well-being factors. Therefore, beside analysing the sleep pattern, we intend to define a daily health score (DHS) which is calculated based on individuals’ physiological parameters—inspired by the concept of Early Warning Scores (EWS) devised in 1997 [52, 53].

The EWSs are simple physiological bedside scoring systems used in hospitals which have been effective guides in early identification of patients at risk of deterioration and in clinical triaging processes [54]. Generally, the clinical response to lower scores include no action or increased monitoring frequency, while higher scores invoke interventions by more senior medical staff or transfer to intensive care units. Despite the maturity of EWS, there is still a lack of agreement on what constitutes an ideal EWS system as each system is calibrated to a different demographic—as such, there have been a few national standardisation modification attempts, e.g. NEWS2 in the United Kingdom [55]. In the TIHM project, the target demographic is the dementia population for which, to the best of our knowledge, there are no special EWS systems in the literature. Additionally, the frequency of monitoring of physiological parameters in TIHM is at most twice daily as opposed to traditional EWS where monitoring frequency may be continuous (e.g. intensive care units). Therefore, we propose a daily scoring system that adapts the concept of existing EWS systems. In this regard, DHS is calculated based on the daily physiological parameters collected in the TIHM project, e.g. body temperature, blood pressure, blood oxygen level, and heart rate, see Table 1.

**Table 1:** Description of TIHM daily health score, adapted from NEWS2 [55].

	Score						
Physiological parameter	3	2	1	0	1	2	3
Systolic blood pressure (mmHg)	≤90	91-100	101-110	111-219			≥220
Pulse (per minute)	≤40		41-50	51-90	91-110	111-130	≥131
Temperature (°C)	≤35		35.1-36	36.1-38	38.1-39	≥39.1	
SpO_2_ (%)	≤91	92-93	94-95	≥96			

The EWS has typically been implemented as part of traditional paper observation charts, where the requirement for scores to be calculated manually necessitated the use of simple scoring algorithms. In addition, storage of data on manually captured and paper-based data has been a barrier to collection of large datasets for score derivation and validation [53]. On the other hand, in TIHM, we employ an automated DHS analysis system to create a continuous history of DHS for each participant and calculate his/her adaptive confidence interval as:

$$\begin{array}{c} \Delta =\mu \pm \xi \frac{\sigma }{\sqrt{d}} \end{array}$$ (4)

in which *ξ* is the confidence coefficient, *d* is the number of days and *μ* and *σ* are the mean and standard deviation of DHS values over *d* days. In this study *d* = 14, meaning that we calculate a personalised Δ for each participant according to his/her physiological readings over the past 14 days. Given this information, we not only monitor the daily value of DHS for participants, but also detect if they are exceeding their personal thresholds. Therefore, once the iForest algorithm detects an abnormal day for an individual, his/her DHS and sleep pattern will be analysed to provide complementary information about the health and well-being conditions of the same day.

## Results and discussions

In this section, we evaluate and compare performances of both supervised and unsupervised UTI prediction models. The algorithms are integrated within the TIHM for Dementia project where analysis is performed based on the data collected for 53 participants (those with minimum of 30 days participation). We have adopted the most commonly used *k*-fold cross-validation strategy in order to optimise, test and train our proposed algorithms. In case of one-class SVM model, we have been provided with annotated datasets by the clinicians. To avoid over-fitting, we have performed a grid search *σ* and *ν* in combination with 5-fold cross-validation for one-class svm model. The selected parameters are found out to be *σ* = 10^−2^ and *ν* = 0.05.

In case of NMF algorithm, we used a subset of three months of data for parameter selection and optimisation, and we used the other three months to blind test the outcomes of our algorithm. During the parameter optimisation stage, *k* has been chosen to be 5, and in all folds NMF was initialised by generating 10 random matrices (*W* and *H*) so that the factorisation with the smallest Euclidean distance between *V* and (*WH*) was chosen for initialisation. To seek a low rank approximation of matrix *V*, we have calculated root-mean square residual error for subspaces between 1 to 6. Since we are computing this low-rank approximation on the data of each individual participant we found out that for most of the cases the residual attain a minimum for a subspace choice of 3 or 4.

### Urinary tract infection

Generally, accuracy measuring the incidence of UTI in the elderly population is challenging due to inconsistent criteria and guidelines. In this study, we have analysed around 680 data matrices on average for every participant that corresponds to their six-hour activity view. The data matrices serve as input to our unsupervised UTI detection algorithm that in turns raise an alert if an anomalous activity pattern is detected. The generated UTI alerts have been assessed by a group of healthcare practitioners (i.e. the monitoring team) who would contact participants and/or their caregivers to confirm whether the generated notifications were reported correctly. These alerts were validated as *True* only if a physician has diagnosed it as positive via urinalysis test, otherwise, labelled as *Not-validated*. It should be mentioned that *Not-validated* alerts were not necessarily false detections, as in some cases the detected UTI alert was suspected to be true, however, it was not confirmed due to the lack of urinalysis test (e.g. when participants were away or did not visit their general practitioner). Therefore, these cases, although suspected, were all labelled as *Not-validated* (rather than *True* or *False*).

The above procedure was used to define the number of truly validated (TV) and not-validated (NV) alerts detected via the proposed algorithm. However, as our participants live in their own homes and due to the fact that we do not have access to their private health records, there was no information regarding the number of true negatives or true positives within this study. Therefore, we have used a validation ratio (*ρ*_val_) and prevalence (*ρ*_prev_) measures to analyse the results of our algorithms, see Eq 5.

$$\begin{array}{c} \begin{array}{c} \rho_{\text{val}}=\frac{\text{TV}}{\text{TV}+\text{NV}},\rho_{\text{prev}}=\frac{\sum \text{Condition}\;\text{Positive}}{\sum \text{Total}\;\text{Population}} \end{array} \end{array}$$ (5)

As shown in Table 2, the supervised UTI model that we have used as a baseline has achieved a validation ratio of 4%. The total number of alerts generated by this model is 153 among which 147 were labelled as NV. In comparison, the unsupervised model that we have developed in this work has achieved a validation ratio of 14% with total number of 35 NVs, which is significantly lower than the supervised model. “Although the overall validation ratios (averaged over all participants) for both models have not been high, we have had two participants for whom our algorithms generated only one UTI alert throughout the time frame of the study and there has not been any false/not-validated alerts (meaning that individual validations rates for these two participants were 100%).” Albeit, given the low prevalence (*ρ*_prev_ = 11%) and high number of not-validated alerts for other participants, the overall rates have declined. It should be mentioned that our solutions utilise the data collected from uncontrolled real-world environments, and we investigated that several alerts were mainly generated due to the presence of guests and unexpected pet movements.

**Table 2:** Performance of supervised and unsupervised UTI detection models.

Participants: 48% women (80.5 ± 5.95) and 52% men (81.57 ± 6.23)				
UTI Model	#TV	#NV	*ρ*_val_	*ρ*_prev_
Supervised	6	147	0.04	11%
Unsupervised	6	35	0.15	11%

According to literature [56], the prevalence of a systematic bacteriuria for women (aged 65-90) and men (aged >65) ranges between 6-16% and 5-21%, respectively. In particular, [57] reported that the incidence of UTI for women and men (over the age of 85) is 13% and 8%, respectively. Given these statistics and the age distribution of our participants, i.e. 48% women (80.5 ± 5.95) and 52% men (81.57 ± 6.23), the calculated prevalence *ρ*_prev_ = 11% (for the trial period of six months) falls within the expected range of the literature [56, 57].

Since the prevalence of UTI in this study is as low as 11%, one option to improve the validation ratio was to increase the number of generated alerts as for larger samples, the validation ratio would shift towards the mean of the distribution. However, in a healthcare system, this would increase the workload by a large magnitude as well increasing the caregivers’ burden. Therefore, our solution focused on decreasing the number of NV alerts per day, while maintaining the number of TV alerts. There are several reasons why the proposed approach has a lower number of NVs compared with the supervised model. Firstly, our pattern decomposition approach evaluates a six-hourly activity pattern in contrast to hourly pattern used by the supervised method. This makes it less sensitive to sudden rise in the movement activity, either by guests or pets. Secondly, the supervised approach relies only on the frequency of bathroom usage and the body temperature values to make a prediction. On the other hand, the unsupervised approach discovers time dependant patterns which are more effective in profiling participants’ routine activities. In addition to bathroom usage, agitation, confusion and irritability are also symptoms of UTI. The unsupervised model generates an alert only if there is a significant deviation from the routine patterns in conjunction with physiological changes. This has led to an improvement in validation ratio as well as reduction in the number of NV alerts.

#### A case study of behavioural anomaly as a symptom of UTI

**Fig 2:**
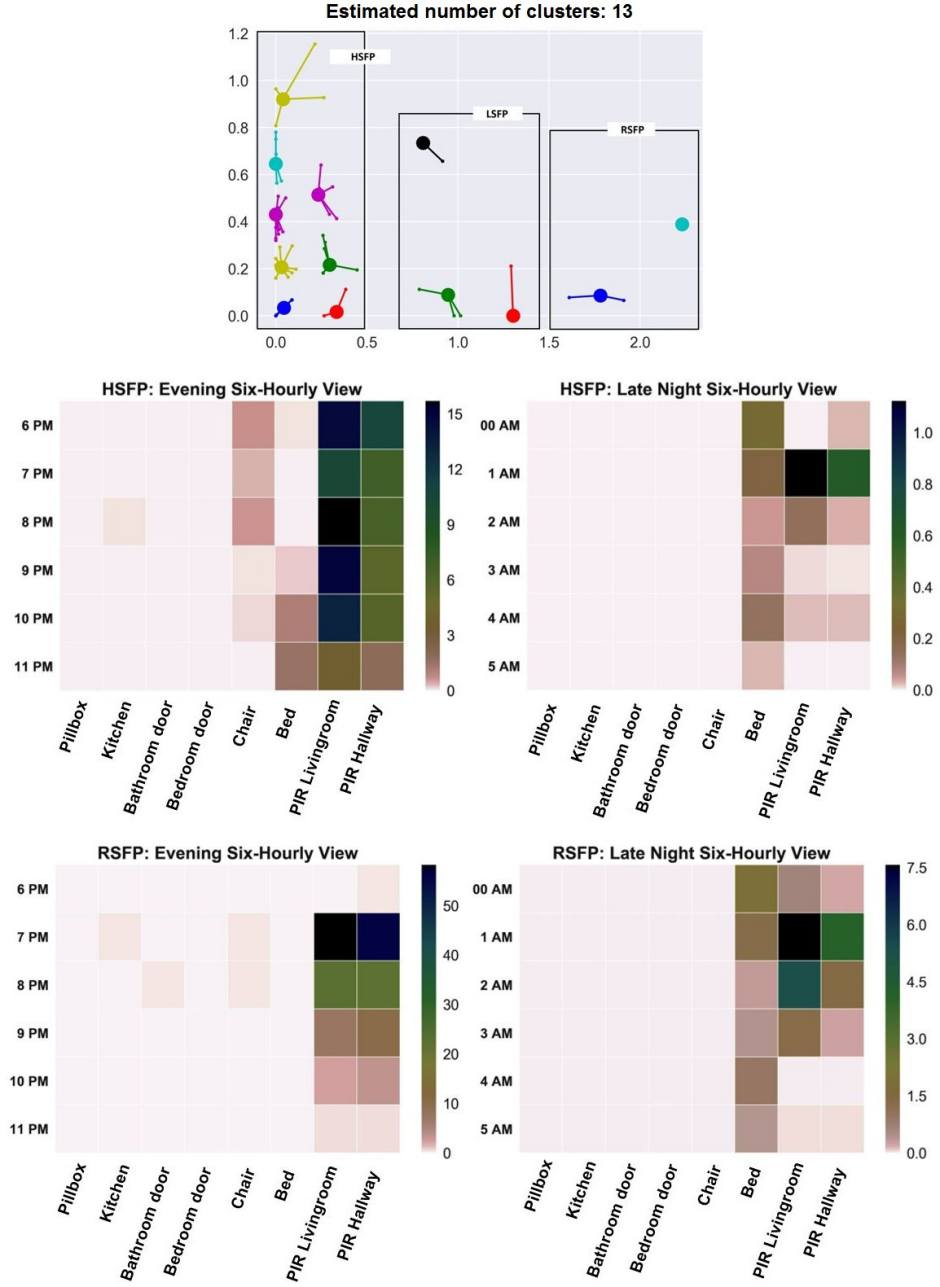
Cluster categorisation of P1 SFPs (top); visualisation of six-hour SFP data matrix belonging to HSFP category (middle) and visualisation of six-hour SFP data matrix belonging to RSFP category (below).

In this particular case, our proposed unsupervised UTI prediction model has generated an alert for an 87 year old male participant, we refer to as P1. A UTI alert has been generated since the decision fusion block within the predictive model has received two alerts in a time window of 12 hours: a rise in body temperature and the anomaly detection module flagged an activity pattern as of belonging to RSFP category. As discussed in Section ‘Unsupervised Early Detection Model’, the activity routine of P1 has been learned in an unsupervised fashion. As shown in Fig 2 (top), the learned profile is then used to classify the real-time data and subsequently an alert is raised if the observed SFP pattern belongs to an RSFP category. In Fig 2 (middle), we present a visualisation of P1’s evening and late evening activity patterns that belongs to HSFP category. It can easily be inferred that the six-hour window of the evening activity is quite different from the late night window. This is expected since P1 is most likely to be asleep in the late night and therefore has lower activity counts for all of the eight viewpoints throughout the six hours. As time of day progresses from 6:00PM to 11:00PM, we observe a clear decreasing pattern of activity counts particularly in the living room and hallway columns. Likewise, this is also true for the chair and kitchen activity.

However, the SFP patterns in the RSFP category exhibit an unusual activity behaviour for the evening and late night time categories, as shown in Fig 2 (below). For example, the activation frequency of sensors in the hallway and the living room belonging to HSFP category has a range between 0 to 15 and 0 to 1 in the evening and late night, respectively. In contrast, the six-hourly patterns belonging to RSFP category shows a much higher activation frequency within a range of 0 to 50 and 0 to 7.5 for evening and late night time categories, respectively, as shown in Fig 2 (middle). Upon visualizing the six-hourly SFP pattern within RSFP category, it can be easily inferred that that P1 is up and wandering in the late night which deviates from its normal routine. Therefore, as soon as RSFP pattern is detected an alert has been triggered to notify the decision block that an anomalous behaviour has been detected. The decision block has raised a UTI alarm since in the last 12 hours, the change detection block has also reported a rise in the body temperature. The UTI alert predicted for P1 by the proposed algorithm was also validated by the clinical team as *True* after P1 undertook a urine culture. The early detection of UTI has allowed P1 to receive an early treatment as he would with the normal healthcare routine.

### Daily pattern analysis

Prior to the integration of sleep pattern information into the daily pattern analysis, it was essential to evaluate the performance of sleep disturbance detection algorithm. As such, we have collected optional self-reports from our participants and validated our results against their reports. Notice that self-reports are generally subjective and might be unreliable due to human factors. In addition, it is not guaranteed that all participants provide daily reports for a long period of time, especially those with dementia. Therefore, we have used the proposed algorithm as the principal approach to provide continuous and objective data, and we used the available self-reports to enhance and evaluate the performance of our algorithm.

In this regard, a total of 50 days were included for 28 participants (only those who provided self-reports for sleep analysis). For each participant, a set of 30 days were considered as the training data to calculate the deviation boundaries, and the other 20 days were analysed as the test data and labelled as either normal or disturbed pattern. Days during which participants were away at night or slept less than three hours, or the data was not collected due to technical issues were excluded from analysis. The results of our proposed algorithm were then validated versus the available self-reports and sensitivity (*ρ*_sen_), specificity (*ρ*_spe_), and overall accuracy (*ρ*) were calculated to quantify the performance. The confusion matrix and quantified evaluation is shown in Table 3.

$$\begin{array}{c} \rho_{\text{sen}}=\frac{\text{TP}}{\text{TP}+\text{FN}},\;\rho_{\text{spe}}=\frac{\text{TN}}{\text{TN}+\text{FP}},\;\rho =\frac{\text{TP}+\text{TN}}{\text{TP}+\text{FP}+\text{FN}+\text{TN}} \end{array}$$ (6)

**Table 3:** Confusion matrix (left) and numerical evaluation (right) of automated sleep analysis (ASA) algorithm vs. self-reports (SR) for 28 participants.

		ASA		ASA	
		Disturbed	Normal	Evaluation	Values
SR	Disturbed	25	9	*ρ*_sen_	0.73
	Normal	19	140	*ρ*_spe_	0.88
	Not validated	24	166	*ρ*	0.85

**Fig 3:**
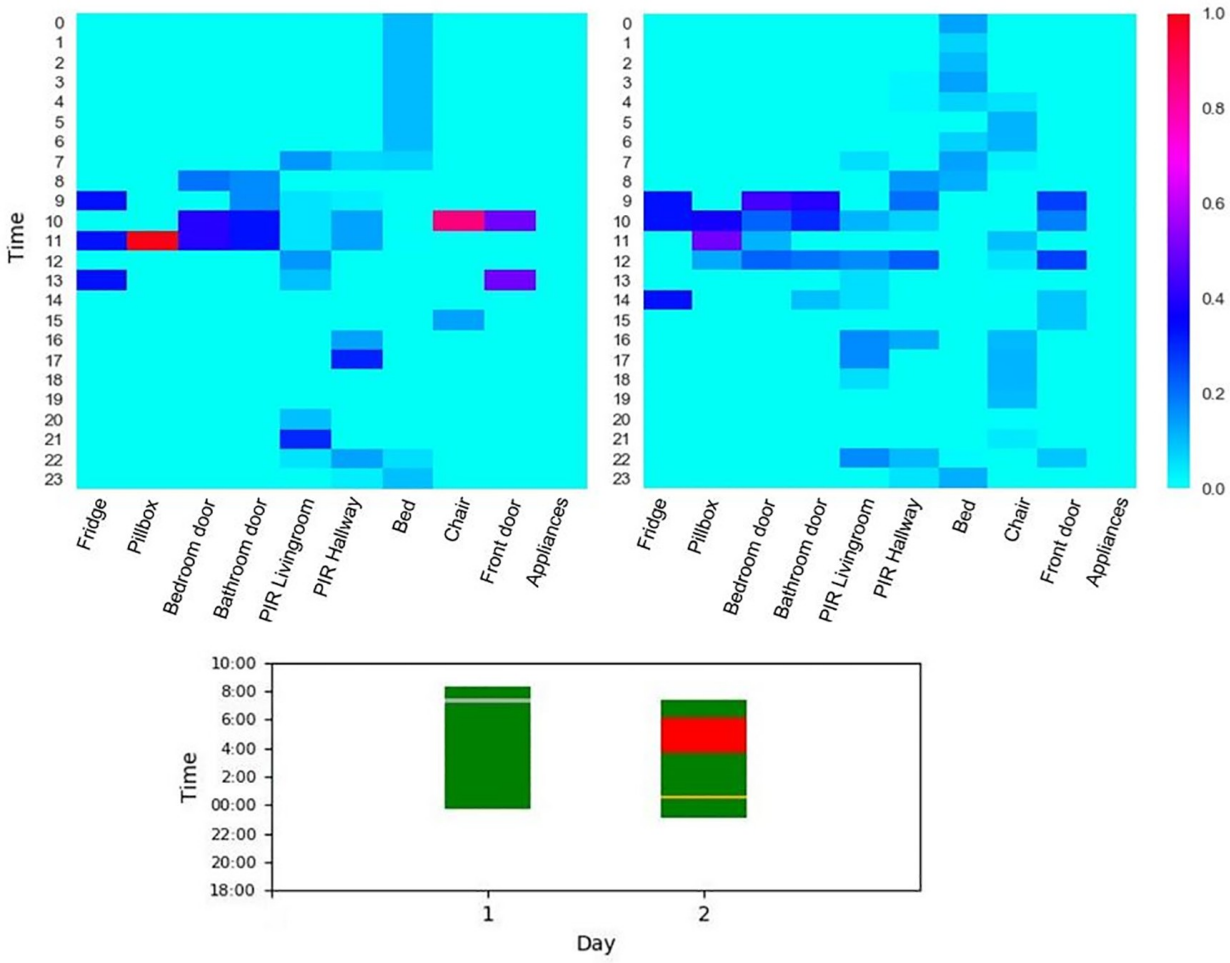
Demonstration of aggregated data collected from an individual’s home for two test days, a normal day (top) versus an abnormal one (middle) and their corresponding night-time sleep pattern (below). The data is aggregated in one hour interval and normalised to ensure that the activity level of each sensor is ranged between 0 to 1.

As shown in Table 3, the proposed algorithm identified night-time sleep disturbances with overall accuracy of 85%. Given that this algorithm is designed based on passive sensors without any occupancy constraint, it provided reasonable performance for uncontrolled real-world environment, i.e. individuals’ homes. As an illustrative example, Fig 3 represents the normalised aggregated data collected from a participant’s home for a normal day (top) versus an abnormal day (middle). As shown, s/he was wandering during the night of the abnormal day where this was also detected by the sleep disturbance algorithm—providing meaningful information about the detected anomaly.

## Conclusions and future work

Recent advancements in medical/clinical technologies have increased the quality of health in many parts of the world. However, long-term conditions such as dementia, diabetes and mental health still require large healthcare resources and support. In this regard, we have introduced a systematic approach to provide more effective and preventative care for people with long-term conditions, and in particular for those with dementia who live in their own homes. We have focused on two key aspects of dementia care including early detection of Urinary Tract Infection (UTI) and identification of changes in daily activity patterns.

UTI is one of the most common reasons of hospital admissions in people with dementia. If a UTI is detected at early stages, it can be resolved by taking antibiotics; however, remaining undiagnosed, a UIT can cause major health issues resulting in hospital admissions. We have designed the UTI detection algorithm using a Non-negative Matrix Factorisation (NMF) method to create a latent space representation of the environmental and physiological data. We have compared our proposed solution with a baseline algorithm that uses a binary support vector machine (SVM) classifier. The results have shown that our unsupervised NMF model outperforms the baseline model, while reducing the number of false positive alerts. The proposed algorithm is especially effective in our current trial in which we do not have access to labelled data collected from a similar setting. This is due to the fact that the combination of environmental and physiological markers have not been previously used for detecting UTIs. While the current precision of the algorithm is not high, we can enhance the performance of the work by collecting more data and employing hybrid and online learning models.

In our previous work [18] we have shown that daily activity patterns are good indicators of general health and well-being of people with dementia. In this study, we have developed a more adaptive solution which is more robust to noisy data and transient changes. The current pattern analysis algorithm uses an Isolation Forest (iForest) algorithm to process the (near-) real-time sensory observations. Most of the existing and dominant solutions in this domain analyse the sequence of daily activities to learn the common patterns in a probabilistic graph model and identify the anomalies by calculating deviations from the learned graph model. The iForest model, however, performs based on a holistic view of daily activities rather than their sequences. This provides more robustness in analysing the noisy environmental sensory data and offers a solution that is more adaptive to subtle changes in the data, while more robust in dealing with transient changes.

The future work will focus on developing a risk awareness score for our integrated solution. We will also extend our work in monitoring and providing healthcare support for other long-term conditions. This will also require extending the machine learning algorithms by combining expert knowledge (captured as semantic knowledge and reasoning rules) with analytical learning models. The work will also focus on combining deep learning models with probabilistic machine learning techniques (e.g. markov chains) to create more adaptive and enhanced models to learn from large volumes of data that which is being collecting within our trial.

## Supporting information

S1 Algorithm Decomposition-based profiling of user behaviour. (PDF)

S2 Algorithm Cluster categorisation. (PDF)

S3 Algorithm Two level rule-based algorithm to analyse environmental data and extract night-time sleep pattern. (PDF)
