# Supplementary material for: Machine learning methods for detecting urinary tract infection and analysing daily living activities in people with dementia
Source: PLoS One. 2019 Jan 15;14(1):e0209909. doi: 10.1371/journal.pone.0209909 (PMC6333356; doi:10.1371/journal.pone.0209909)
Supplement: S1 Algorithm — (PDF) [file pone.0209909.s001.pdf]

Supporting information

S1 Algorithm: Decomposition-based Profiling of User Behaviour

Input: Matrix of column data V, desired dimension of feature space r

1 Factor $V \approx WH$, where W is $m \times r$ and H is $r \times n$.

2 Use Affinity propagation clustering Algorithm [**?**], to cluster the columns of H, that are the coordinates of the data points after projection into a r-dimensional space.

Output: Cluster of data points that represents the SFPs.
