# Supplementary material for: Machine learning methods for detecting urinary tract infection and analysing daily living activities in people with dementia
Source: PLoS One. 2019 Jan 15;14(1):e0209909. doi: 10.1371/journal.pone.0209909 (PMC6333356; doi:10.1371/journal.pone.0209909)
Supplement: S2 Algorithm — (PDF) [file pone.0209909.s002.pdf]

Supporting information

S2 Algorithm: Cluster Categorisation

Input: A one-dimensional array A consisting of M elements, each representing the count of SFPs within each cluster obtained by Algorithm I

Perform median absolute deviation (MAD) analysis to calculate a MAD score.

Use the following equation to assign categories:

$$X - D \times MAD < x_i < X + D \times MAD \tag{1}$$

$$\frac{x_i - X}{MAD} > \| \pm D\| \text{ where } D \text{ is the rejection criteria} \tag{2}$$

Output: Based on the values of D, assign a cluster to either a HSFP, LSFP or RSFP category.
