# Supplementary material for: Machine learning methods for detecting urinary tract infection and analysing daily living activities in people with dementia
Source: PLoS One. 2019 Jan 15;14(1):e0209909. doi: 10.1371/journal.pone.0209909 (PMC6333356; doi:10.1371/journal.pone.0209909)

Supporting information

S3 Algorithm: Two level rule-based algorithm to analyse environmental data and extract night-time sleep pattern

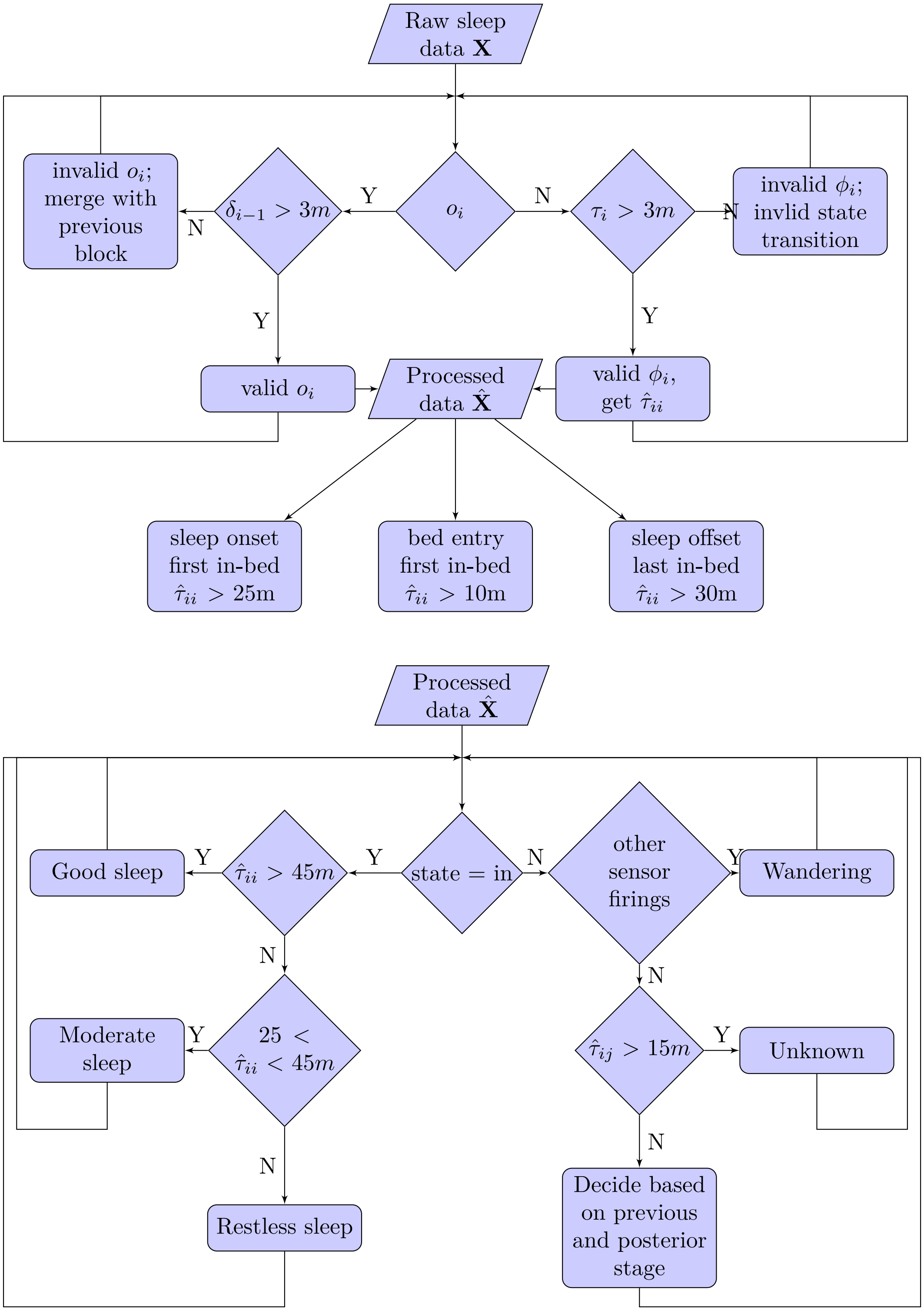

Supplement: S3 Algorithm — (PDF) [file pone.0209909.s003.pdf]
